# Long-term administration of CU06-1004 ameliorates cerebrovascular aging and BBB injury in aging mouse model

**DOI:** 10.1186/s12987-023-00410-x

**Published:** 2023-02-01

**Authors:** Hyejeong Kim, Minyoung Noh, Haiying Zhang, Yeomyeong Kim, Songyi Park, Jeongeun Park, Young-Guen Kwon

**Affiliations:** 1 Department of Biochemistry, College of Life Science and Biotechnology, Yonsei University, Seoul, 03722 Korea; 2 CURACLE Co., Ltd., Seoul, 06694 Korea

**Keywords:** CU06-1004, Blood–brain barrier, Aging, Brain microvascular endothelial cell (BMEC), Reactive oxygen species (ROS), Cerebrovasculature, Inflammation, Neurodegenerative disorders

## Abstract

**Background:**

Age-related changes in the cerebrovasculature, including blood–brain barrier (BBB) disruption and vascular dementia, are emerging as potential risks for many neurodegenerative diseases. Therefore, the endothelial cells that constitute the cerebrovasculature may play key roles in preventing brain injury. Our previous study showed that CU06-1004, an endothelial cell dysfunction blocker, prevented vascular leakage, enhanced vascular integrity in ischemic reperfusion injury, and promoted the normalization of tumor vasculature. Here, we evaluated the effects of CU06-1004 on age-related cerebrovascular functional decline in the aged mouse brain.

**Results:**

In this study, we investigated the protective effects of CU06-1004 against oxidative stress–induced damage in human brain microvascular endothelial cells (HBMECs). HBMECs were treated with hydrogen peroxide (H_2_O_2_) to establish an oxidative stress–induced model of cellular injury. Compared with H_2_O_2_ treatment alone, pretreatment of HBMECs with CU06-1004 considerably reduced oxidative stress–induced cytotoxicity, reactive oxygen species generation, senescence-associated β-galactosidase activity, senescence marker expression, and the expression levels of inflammatory proteins. Based on the observed cytoprotective effects of CU06-1004 in HBMECs, we examined whether CU06-1004 displayed protective effects against cerebrovascular aging in mice. Long-term administration of CU06-1004 alleviated age-associated cerebral microvascular rarefaction and cerebrovascular senescence in the aged mouse brain. CU06-1004 supplementation also reduced the extravasation of plasma IgG by improving BBB integrity in the aged mouse brain, associated with reductions in neuronal injury. A series of behavioral tests also revealed improved motor and cognitive functions in aged mice that received long-term CU06-1004 administration.

**Conclusions:**

These findings suggest that CU06-1004 may represent a promising therapeutic approach for delaying age-related cerebrovascular impairment and improving cognitive function in old age.

**Supplementary Information:**

The online version contains supplementary material available at 10.1186/s12987-023-00410-x.

## Background

The blood–brain barrier (BBB) is a physical barrier composed of brain microvascular endothelial cells (BMECs) that limit the movement of substances from circulating blood into the brain, maintaining brain homeostasis. BMECs in cerebral blood vessels form tight junctions that play important roles in BBB integrity [1, 2].

During aging, various stimuli and environmental factors can cause BMECs to lose their abilities to proliferate, migrate, and repair damage [3, 4]. Damaged BMECs transition into senescence, an irreversible state of growth arrest. The accumulation of senescent cells in organs leads to the release of high levels of inflammatory cytokines, matrix metalloproteinases, and immune regulators, which can induce the development of a senescence-associated secretory phenotype (SASP) in the surrounding microenvironment. SASP development, which can occur even if only 2–3% of endothelial cells become senescent, is thought to be the primary cause of age-related diseases [5, 6]. The induction of senescence among BMECs in the cerebrovascular system could result in BBB disruption, mild cognitive impairment, and vascular dementia, with major implications for the development of cerebrovascular diseases and neurodegenerative disorders [7–9].

Accumulating evidence demonstrates that oxidative stress and inflammation are the primary factors driving cellular senescence [10, 11]. Inflammatory cytokines secreted by senescent cells trigger further inflammation and senescence in surrounding tissues [12]. Additionally, the increased presence of inflammatory cytokines reduces endogenous antioxidative enzyme levels and induces the accumulation of reactive oxygen species (ROS) in tissue [13]. The cerebrovascular system is particularly sensitive to oxidative stress, and the accumulation of ROS can lead to BBB disruptions [13–15]. Many studies have shown that the BBB structure and function deteriorate during aging, leading to increased BBB permeability [16–18]. Disruption of the BBB is associated with the loss of motor neurons, neuroinflammation, and cognitive impairment [19]. However, few pharmaceutical interventions have been identified as therapeutic candidates for preserving BBB functionality and preventing cerebrovascular aging [20–22].

CU06-1004 is a small molecule known to activate the cAMP/Rac/cortactin pathway, strengthening the tight junction barrier in endothelial cells and blocking hyperpermeability [23, 24]. Acute CU06-1004 treatment for ischemia/reperfusion-induced BBB injury reduced cerebral edema and astrocyte end-foot disruption by stabilizing endothelial cell junctions [25]. Based on these previous findings, we investigated the effects of CU06-1004 on age-related cerebrovascular functional decline in the aged mouse brain. To investigate the role and potential molecular mechanisms of CU06-1004 in the aged brain, we used both an in vitro cell model of hydrogen peroxide (H_2_O_2_)-induced oxidative stress injury and an in vivo mouse model of natural aging. Our results showed that CU06-1004 treatment inhibited oxidative stress–induced senescence in HBMECs and reduced inflammation by suppressing nuclear factor-kappa B (NF-κB) signaling. Furthermore, we report the novel finding that long-term oral CU06-1004 administration improves age-associated cerebral microvascular rarefaction in aged mice. Notably, treatment with CU06-1004 increased the expression of tight junction proteins in the endothelial cells of the cerebral microvasculature, which are critical for BBB maintenance. Consequently, CU06-1004 treatment attenuated neuropathological changes in the aged brain. We also found that CU06-1004 treatment rescued cognitive deficits and enhanced muscle function in 23-month-old mice. Our results demonstrate that CU06-1004 effectively ameliorates age-associated cerebrovascular aging and brain injury, suggesting that CU06-1004 has the potential for use as an effective therapy protecting against the development and progression of age-related cerebrovascular diseases.

## Materials and methods

### Drug treatment

CU06-1004 was synthesized as described previously [23]. Briefly, CU06-1004 was synthesized via tetrahydropyran deprotection and subsequent glycosidation with 4,6-*di*-*O*-acetyl-2,3-didieoxyhex-2-enopyran in the presence of an acid. A working solution of CU06-1004 (10 µg/µl) was prepared in dimethyl sulfoxide (DMSO, Sigma, #D2650) for in vitro experiments. For in vivo experiments, CU06-1004 was dissolved in olive oil (Sigma, #O1514) for oral administration. Mice (72 weeks old) were divided into two groups. The old-vehicle group was administered vehicle only (n = 15), and the old-1004 group was administered CU06-1004 (10 mg/kg, n = 15). Both vehicle and CU06-1004 treatments were orally administered 6 days per week using a Zonde needle (100 µl, Jeung Do Bio & Plant Co, #JD-S124) for 6 months (age 18–24 months). No symptoms, such as diarrhea, were observed. However, natural weight loss was observed with increased age in both old-vehicle and old-1004 mice, with no significant difference in body weights between the two aged groups.

### Experimental animals

Male C57BL/6 J mice (72 weeks old) were purchased from Charles River Laboratories Japan (Yokohama, Kanagawa, Japan). Additionally, 6-week-old male C57BL/6 J mice (DBL, Korea) were used as young mice, and 24-month-old male C57BL/6 J mice were used as aged mice. All mice were housed under controlled conditions (24 °C ± 1 °C, 12-h light/dark cycles, 55% humidity, and specific pathogen–free) and provided with free access to food and water. All animal facilities and experiments were performed in accordance with the Korean Food and Drug Administration guidelines. All procedures were approved by the Institutional Animal Care and Use Committee at Yonsei University (permit number: IACUC-A-202010-1154-01).

### Primary cultures of human brain microvascular endothelial cells

Human BMECs (HBMECs) were purchased from ScienCell (Cat. No. 1000) and cultured in endothelial cell growth medium 2 (EGM-2; Lonza, CC-3156) supplemented with the EGM-2 SingleQuots™ kit (Lonza, CC-4176), 20% fetal bovine serum, and 1% penicillin/streptomycin (Cat. No. 0503). Cells were routinely passaged at 80–90% confluency, and cells between passages 3 and 6 were used for experiments. Cells were maintained at 37 °C in a humidified atmosphere containing 5% CO_2_.

### Measurement of cell viability

Colorimetric 3-(4,5-dimetylthialzol-2-yl)-2,5-diphenyltertrazolium bromide (MTT; Thermo Fisher Scientific, #M6494) assay was used to measure cell viability. MTT is reduced to formazan by mitochondrial dehydrogenases, and the absorbance (570 nm) is directly proportional to the viable cell count. HBMECs were seeded into a gelatin-coated 24-well plate at 1 × 10^5^ cells/well and incubated at 37 °C in EGM-2 medium overnight. The following day, the cells were treated with either CU06-1004 or H_2_O_2_. The cells were then washed with 1 × phosphate-buffered saline (PBS) and incubated for 4 h at 37 °C with MTT solution (0.1 mg/ml) to evaluate cell viability. After the 4-h incubation, the MTT solution was removed, and a 50:50 solution of DMSO and ethanol was added (200 µl/well) to solubilize formazan crystals. Absorbance was detected at a wavelength of 570 nm, and cell viability was calculated as a percentage of the absorbance detected from the control cells.

### RNA isolation and reverse transcription–polymerase chain reaction

To perform reverse transcription–polymerase chain reaction (RT-PCR), total RNA was extracted from HBMECs using easy-BLUE™ (iNtRON, #17061). Total RNA was reverse transcribed into cDNA using M-MLV Reverse Transcriptase (Promega Corporation, #M1701) in the presence of oligo(dT) primers and dNTP. The following temperature protocol was used for reverse transcription: Denaturation at 70 °C for 5 min, annealing at 25 °C for 10 min, and extension at 42 °C for 50 min. The following primers were used for PCR: p21, 5′-GCTTCATGCCAGCTACTTCC-3′ (forward), 5′-CCCTTCAAAGTGCCATCTGT-3′ (reverse); p16, 5′-CCTCGTGCTGATGCTACTGA-3′ (forward), 5′-CATCATCATGACCTGGTCTTCT-3′ (reverse); and glyceraldehyde-3-phosphate dehydrogenase (GAPDH), 5′-CCACCCATGGCAAATTCC-3′ (forward), 5′-TCGCTCCTGGAAGATGGTG-3′ (reverse). All results were normalized to GAPDH expression levels.

### Measurement of intracellular reactive oxygen species

The formation of ROS was measured using a ROS-sensitive indicator dye: 2’,7’-dichlorodihydrofluorescein diacetate (H_2_-DCFDA, Invitrogen, #D399). HBMECs were seeded at 1 × 10^4^ cells/well in a black, clear-bottom, 96-well plate containing 100 µl culture medium and incubated at 5% CO_2_ and 37 °C overnight. The following day, HBMECs were starved of media for 2 h and then pretreated with CU06-1004 (10 µg/ml) for 1 h. The media were removed, and the cells were washed twice with PBS, followed by incubation with 100 µM H_2_O_2_ for 2 h to stimulate ROS development. The cells were then incubated with 10 µM H_2_-DCFDA for 30 min at 37 °C. The fluorescent product formation was quantified with a spectrofluorometer at 485/520 nm. The fluorescent cells were then washed twice with PBS and observed using a fluorescence microscope (Microscope, Olympus DX51; Camera, Olympus DP72).

### Senescence-associated-β-galactosidase staining

Samples were fixed with 3.7% formaldehyde for 10 min and washed with cold 1 × PBS for 15 min at room temperature (RT). Samples were washed twice more with PBS and then incubated with senescence-associated β-galactosidase (SA-β-Gal) staining solution [1 mg/ml 5-bromo-4-chloro-3-indolyl-β-D-galactopyranoside (X-gal, MERCK, #B4252), 5 mM potassium ferrocyanide, 5 mM potassium ferricyanide, 150 mM NaCl, 2 mM MgCl_2_, and 0.01% Nonidet-P-40 (NP-40)] for 24 h at 37 °C without CO_2_. After the 24-h incubation, samples were washed with PBS, and the degree of blue color development was used to indicate aging [26]. Staining and imaging were observed under a phase-contrast microscope (Nikon, Japan).

### Quantitative immunofluorescent microscopy of cerebral immunoglobulin G extravasation

The integrity of the BBB was determined by the detection of cerebral perivascular extravasation of the plasma protein immunoglobulin G (IgG), a widely used and established method [27]. To clear blood and remove vascular IgG, 24-month-old mice were anesthetized using avertin (2, 2, 2-tribromoethanol, Sigma Aldrich, #T48402; 250 mg/kg of body weight) and perfused with 0.9% saline solution injected into the apex of the left ventricle. Brain tissues were immersion-fixed in 4% paraformaldehyde for 24 h and immersed in 15% and 30% sucrose each day. The tissues were then frozen in optimal cutting temperature (OCT) compound and stored at − 80 °C. Brain cryosections (25-µm-thick) were placed on Polysine™-coated microscope slides (Leica, #3800050CL). The sections were prefixed in acetone for 30 min at − 70 °C. Non-specific binding was blocked by incubation with 10% goat serum in PBS for 30 min. Sections were incubated with goat anti-mouse IgG conjugated with Alexa 488 (1 mg/ml, 1:50, Invitrogen, #A28175) at 4 °C for 20 h. After washing sections with 0.2% Tris-buffered saline containing Tween 20 (TBST) and 1 × PBS, the sections were mounted with mounting solution (DAKO, #S3023). Immunofluorescent images were acquired using a Zeiss LSM980 confocal microscope (Zeiss, Germany) at 20 × magnification. Images were analyzed by Zen blue software (Zeiss, Germany). Quantification of fluorescence intensity was performed using Photoshop version CS6 (Adobe Systems, San Jose, CA). For each cortical and hippocampal region, 5–6 images were randomly obtained from each brain section, and all images were used for subsequent quantitative analyses.

### Quantitation of IL-6 and TNF-α by enzyme-linked immunosorbent assay

Cardiac puncture was performed to obtain blood samples. Blood was collected in serum-separating tube (Becton Dickinson, # BD365967) and incubated for 30 min at RT. Samples were centrifuged at 201 × *g* for 10 min at RT to obtain murine serum. Serum concentrations of interleukin (IL)-6 and tumor necrosis factor-alpha (TNF-α) were determined using Quantikine ELISA Kit (R&D systems, #M6000B, #MTA00B), according to the manufacturer’s protocol.

### Histology and immunohistochemical analysis

After 6 months of drug administration, 24-month-old mice were anesthetized using avertin (2, 2, 2-tribromoethanol, Sigma Aldrich, #T48402; 250 mg/kg of body weight) and perfused with 0.9% saline solution injected into the apex of the left ventricle. Brain tissue was removed and fixed in 4% paraformaldehyde in PBS (pH 7.4) overnight at 4 °C. Following overnight fixation, brain tissue was incubated in 15% sucrose overnight at 4 °C and then transferred to 30% sucrose at 4 °C until the tissue sank. Fixed tissue was encapsulated in Tissue-Tek OCT embedding medium for 30 min at RT, transferred to an embedding mold filled with OCT, frozen on dry ice, and stored at − 70 °C. Frozen section (25-µm-thick) were cut at − 20 °C, and slides were stored at − 80 °C until stained for immunofluorescence. Sections were prefixed in acetone for 30 min at − 70 °C and air dried. OCT was washed off with running tap water. Sections were incubated in blocking solution for 1 h at RT and then incubated overnight at 4 °C with the following primary antibodies: CD31 (1 µg/ml, 1:200; Abcam, #ab24590), glial fibrillary acidic protein (GFAP; 1:200; Millipore, #MAB360), claudin-5 (0.5 mg/ml, 1:200; Invitrogen, #35-2500), and occludin (0.25 mg/ml, 1:200; Invitrogen, #711500). After incubation, sections were washed three times with 0.2% Triton X-100 in PBS (10 min/wash) and further incubated separately with appropriate 488-conjugated secondary antibody (1 mg/ml, 1:400, Invitrogen, #A28175), 594-conjugated secondary antibody (2 mg/ml, 1:400; Invitrogen, #A21207), and 4′,6-diamidino-2-phenylindole (DAPI; 1 mg/ml, 1:1000, Duolink, #D9542). Stained sections were analyzed using a confocal microscope (LSM 880 META; Carl-Zeiss).

### Western blot analysis

Western blotting was performed as previously described [28]. Briefly, HBMECs were lysed using radioimmunoprecipitation assay (RIPA) buffer (100 mM Tris–Cl, 5 mM EDTA, 50 mM NaCl, 50 mM β-glycerophosphate, 50 mM NaF, 0.1 mM Na_3_VO_4,_ 0.5% NP-40, 1% Triton X-100, and 0.5% sodium deoxycholate) at 4 °C. Sample protein concentrations were quantified using the SMART™ BCA Protein Assay Kit (iNtRON, #21071). Cell lysates (25 µg) were separated by sodium dodecyl sulfate–polyacrylamide gel electrophoresis and transferred to nitrocellulose membranes. Membranes were blocked with 3% bovine serum albumin in 0.1% TBST and probed with primary antibodies. Membranes were then incubated with horseradish peroxidase–conjugated goat anti-rabbit IgG (0.8 mg/ml, 1:1000, Thermo Scientific, #31460) or goat anti-mouse IgG (0.8 mg/ml, 1:1000, Thermo Scientific, #31430) secondary antibodies. β-Actin was used as the loading control. The following primary antibodies were obtained from Cell Signaling Technology and were used at a 1:1000 dilution: phospho–NF-κB inhibitor (IκB)-α (#9242), IκB-α (#9242). The other primary antibodies used were intracellular adhesion molecule 1 (ICAM-1; 200 µg/ml, 1:1000, Santa Cruz Biotechnology, #SC-8439), vascular cell adhesion molecule 1 (VCAM-1; 200 µg/ml, 1:1000, Santa Cruz Biotechnology, #SC-13160), cyclooxygenase 2 (COX-2; 200 µg/ml, 1:1000, Santa Cruz Biotechnology, #SC-376861), and β-actin (1 µg/ml, 1:2000, Thermo Fisher Scientific, #MA5-15739).

### Behavior tests

#### Wire hang test

The wire hang test was conducted to evaluate mouse forelimb strength. The apparatus consisted of a stainless-steel wire (90 cm in length, 2 mm in diameter) secured horizontally between two vertical stands, 30 cm above a soft, padded surface. The wire hang test was conducted when mice were 23 months of age. The mouse was forced to grasp the central position of the wire with its forepaws, and the time until the mouse fell from the wire to the pad was measured. When the time reached 150 s, the mouse was released from the wire, and the time was recorded as 150 s. The trial was repeated three times for each mouse, and the average value across all three trials was used for evaluation. Mice were allowed to rest for 3 min between consecutive attempts.

#### Rotarod test

The rotarod test was conducted to assess motor coordination. Mice were placed on a rotarod device (Four Lane Rotarod; Ugo Basile, Italy, #MSW-007) consisting of a rod rotating at an accelerating speed that the mice must balance on. If the mouse loses its balance and falls, the rod automatically stops and records the time to fall and the rotating speed at the time of the fall. Prior to the first test, the mice were habituated to the testing system until they were able to stay on a rod rotating at a constant speed of 2 rpm for approximately 1 min. During testing, each animal was placed on the apparatus three times for 300 s per trial. The initial rotation speed was 4 rpm and increased to 50 rpm over 300 s. When the mouse fell, the session was over, and the Ugo Basile program stopped the timer [29].

### T-maze alteration

Spatial working memory was assessed using a simple T-maze test [30]. Each trial consisted of a sample run and a choice run. During the sample run, one of the goal arms was blocked, forcing the mouse to enter the other goal arm (e.g., the left arm). A 30-s interval separated the sample run from the choice run, and a 30-s interval was used between trials. During the choice run, both arms were open, and the mouse was able to choose either arm. Even without a reward, driven by curiosity, mice usually selected the previously unvisited arm (e.g., the right arm). The animal was considered to have made a correct choice (+) if it visited the previously unsampled arm and an incorrect (−) choice if it visited the previously sampled arm. A total of 10 free choices made by each mouse were measured, and the percentage of correct arm choices during the choice trials was calculated. Each arm of the T-maze was cleaned between sessions using ethanol to remove any olfactory cues, which may have affected the behavior of the next mouse tested.

### Statistical analysis

Data were analyzed using repeated-measures one-way analysis of variance, followed by a post hoc Tukey’s multiple comparison test. Data are presented as the mean ± standard deviation (SD) or as the mean ± standard error of the mean (SEM). *P* -value less than 0.05 was significant. All statistical analyses were performed using GraphPad Prism version 8 (GraphPad Software, San Diego, CA, USA).

## Results

### CU06-1004 reduces H_2_O_2_-induced inhibition of HBMEC growth

**Fig. 1:**
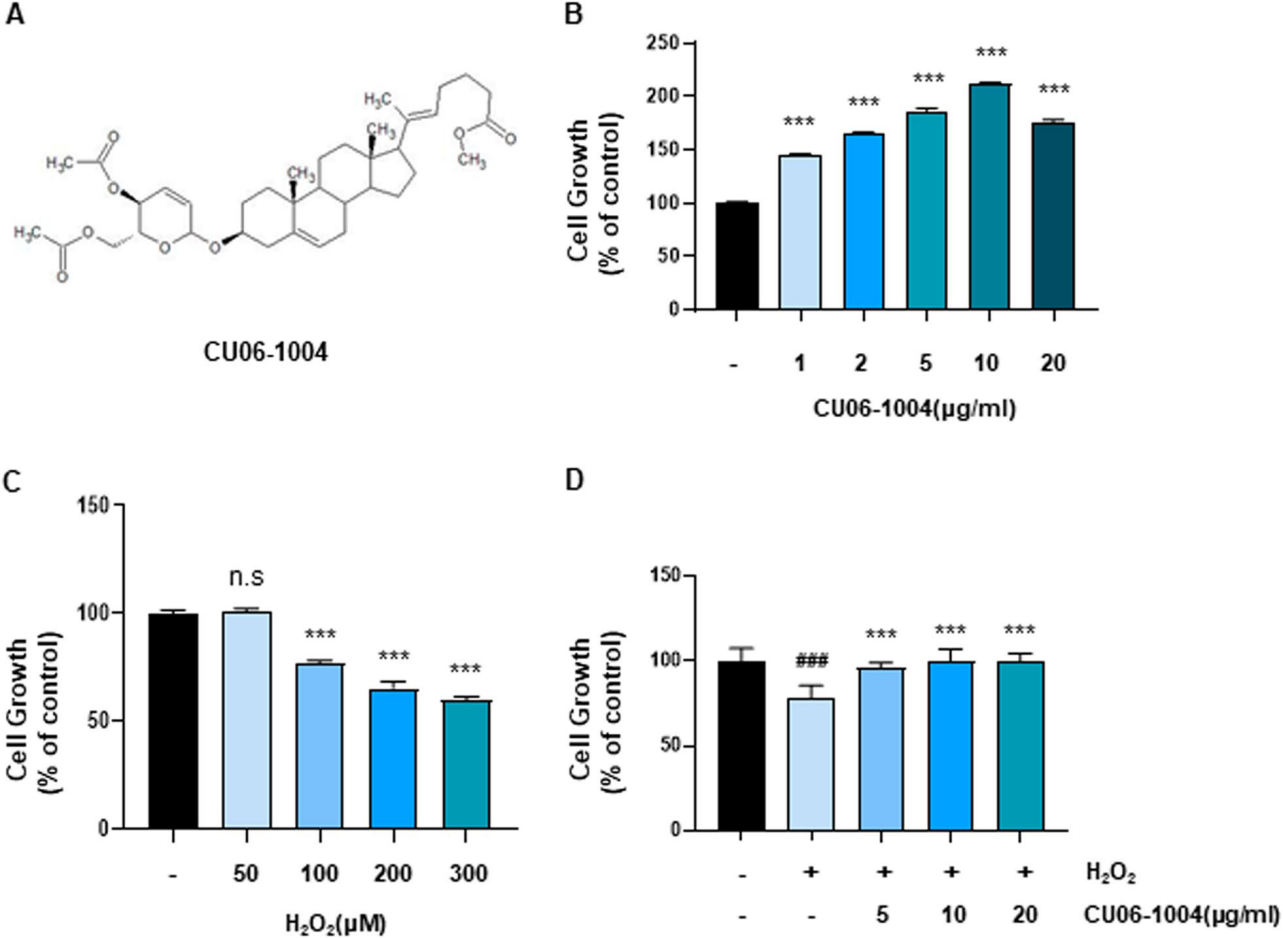
CU06-1004 reduces H_2_O_2_ -induced inhibition of HBMEC growth. **A** Chemical structure of CU06-1004. **B** Human brain microvascular endothelial cells (HBMECs) were incubated with increasing concentrations of CU06-1004 (1, 2, 5, 10, 20 µg/ml) for 48 h. Cell viability was determined using 3-(4, 5-dimethylthiazol-2-yl)-2, 5-diphenyltetrazolium bromide (MTT) assay. **C** Cell viability of HBMECs treated with increasing concentrations of H_2_O_2_ for 24 h. Cell viability was determined using MTT assay. **D** HBMECs were pretreated with CU06-1004 (5–20 µg/ml) for 1 h before 100 µM H_2_O_2_ exposure. After 24 h, cell viability was determined using MTT assay. All data were analyzed with one-way analysis of variance followed by Tukey’s multiple comparisons test. B-C. ***P < 0.001 vs. control. n.s., not significant. D. ^**###**^*P* < 0.001 vs. control. ***P < 0.001 vs. H_2_O_2_. Results are presented as the mean, and error bars represent the standard error of the mean

Prior to investigating the protective properties of CU06-1004 (Fig. 1A) against H_2_O_2_ treatment, the cytotoxic potential of CU06-1004 was examined in HBMECs. The MTT assay indicated no cytotoxic effects for CU06-1004 treatment at concentrations ranging from 1 to 20 µg/ml (1, 2, 5, 10, and 20 µg/ml). Conversely, CU06-1004 treatment significantly increased HBMEC growth in a dose-dependent manner (Fig. 1B). Further MTT analyses revealed that treatment with 50 µM H_2_O_2_ did not inhibit HBMEC growth, whereas treatment with 100 µM H_2_O_2_ significantly inhibited HBMEC growth (Fig. 1C). However, CU06-1004 pretreatment effectively prevented the H_2_O_2_-induced inhibition of cell growth (Fig. 1D). These results demonstrate that CU06-1004 pretreatment is able to prevent the H_2_O_2_-induced inhibition of HBMEC growth.

### CU06-1004 inhibits H_2_O_2_-induced ROS generation and alleviates the inflammatory response of HBMECs

**Fig. 2:**
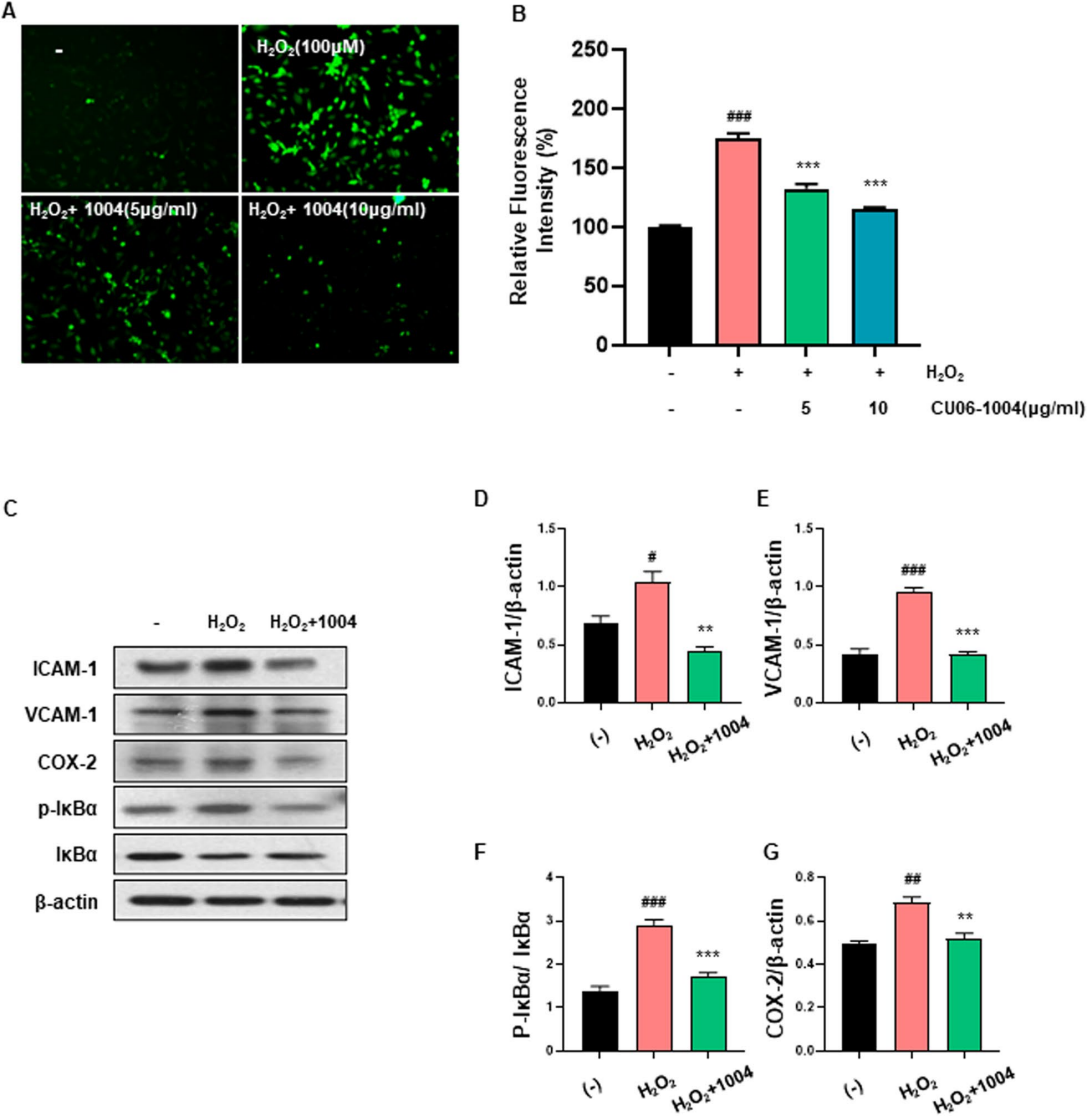
CU06-1004 inhibits H_2_O_2_-induced reactive oxygen species generation and alleviates the inflammatory response in HBMECs. **A** Representative fluorescent images indicating reactive oxygen species (ROS) production in human brain microvascular epithelial cells (HBMECs). HBMECs were pretreated with 5 and 10 μg/ml CU06-1004 for 1 h, followed by incubation with 100 μM H_2_O_2_ for 2 h. Cells were then labeled with 2’, 7’-dichlorodihydrofluorescein diacetate (H_2_-DCFDA) to measure ROS production. **B** The levels of ROS were detected by fluorescence microscopy with H_2_-DCFDA as the fluorescent probe. Quantitative analysis was performed by measuring the fluorescence intensity relative to control cells. **C** CU06-1004 suppressed ROS-mediated nuclear factor-kappa-B (NF-κB) activation in HBMECs. HBMECs were pretreated with CU06-1004 for 1 h and treated with H_2_O_2_ for 6 h. **D**–**G** Quantitative analysis of intracellular adhesion molecule 1 (ICAM-1), vascular cell adhesion molecule 1 (VCAM-1), and cyclooxygenase-2 (COX-2) expression levels normalized to β-actin levels. Phosphorylated NF-κB inhibitor (p-IκBα) expression levels are normalized to total IκBα levels. All expression levels were evaluated by western blotting. All data were analyzed with one-way analysis of variance, followed by Tukey’s multiple comparison test. ^**#**^*P* < 0.05, ^**###**^*P* < 0.001 vs. control. **P < 0.01, ***P < 0.001 vs. H_2_O_2_. Results are presented as the mean, and error bars represent the standard error of the mean

To elucidate the possible mechanisms through which CU06-1004 prevents the H_2_O_2_-induced inhibition of HBMEC growth and determine the effect of CU06-1004 on H_2_O_2_-induced oxidative stress, we used H_2_-DCFDA, a cell-permeable dye that fluoresces upon oxidation by ROS. HBMECs treated with H_2_O_2_ for 2 h showed significantly enhanced ROS generation compared with untreated HBMECs. However, pretreatment with CU06-1004 for 1 h significantly inhibited the H_2_O_2_-induced increase in ROS generation in a dose-dependent manner (Fig. 2A, B). Many studies have determined that oxidative stress induces an inflammatory response, either directly or indirectly, and oxidative stress and inflammation are primary mechanisms related to the onset of age-related vascular endothelial dysfunction [31–33]. Therefore, to investigate whether CU06-1004 pretreatment was able to alleviate the inflammatory response induced by oxidative stress, we examined the expression of inflammatory proteins in H_2_O_2_‑treated HBMECs. As shown in Fig. 2C–G, CU06-1004 pretreatment effectively inhibited the H_2_O_2_-induced expression of ICAM-1, VCAM-1, and COX-2, an inflammation-mediating enzyme. Furthermore, the transcription factor NF-κB is considered a major mediator of the inflammatory response, and the level of phosphorylated IκBα, an indicator of NF-κB activity, was significantly reduced in HBMECs pretreated with CU06-1004. Together, these results suggest that CU06-1004 pretreatment exerts a protective effect against ROS-induced damage by inhibiting the inflammatory response in HBMECs.

### CU06-1004 reverses H_2_O_2_-induced senescence in HBMECs

**Fig. 3:**
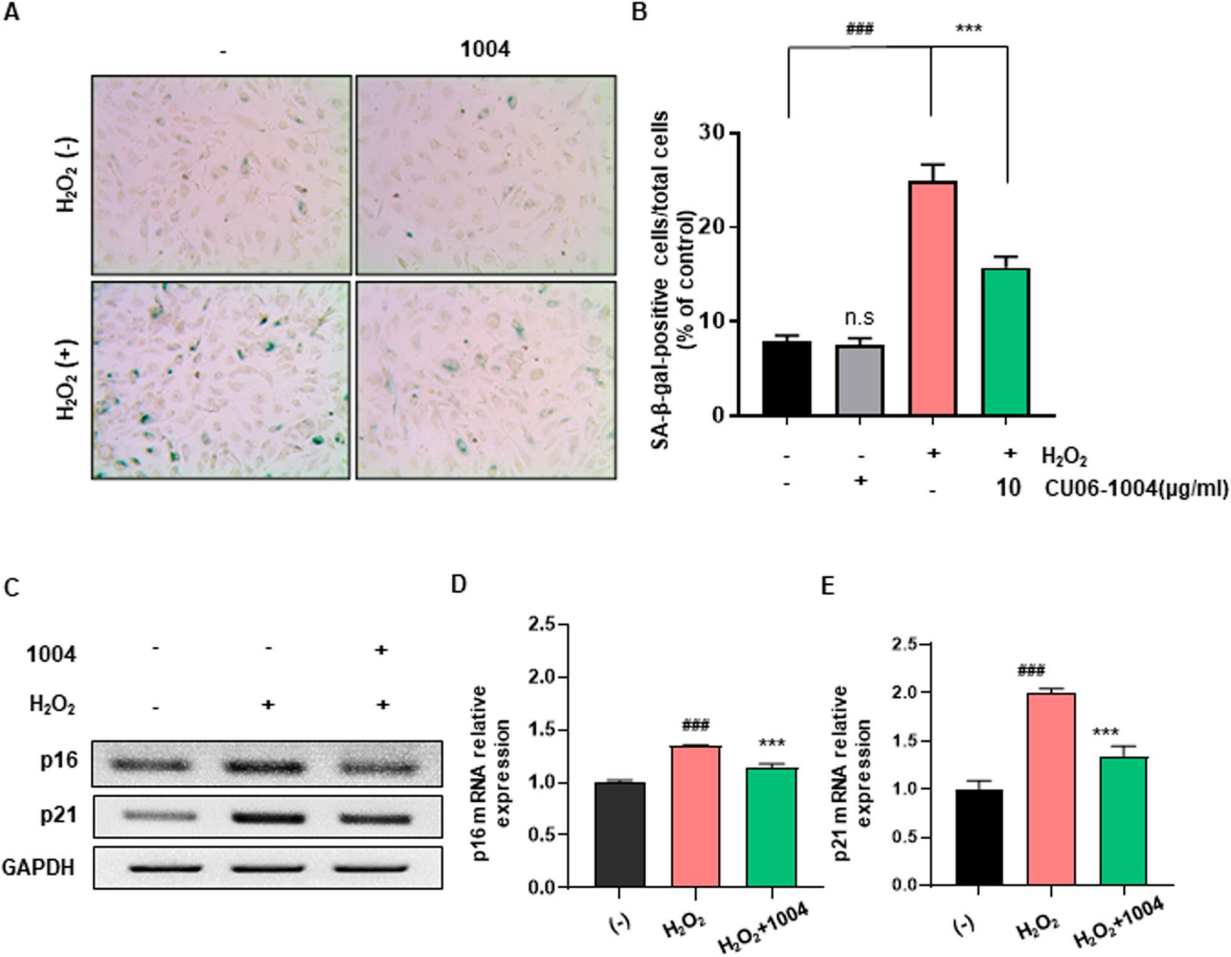
CU06-1004 reverses H_2_O_2_-induced senescence in HBMECs. **A** Senescence-associated β-galactosidase (SA-β-Gal) staining in human brain microvascular endothelial cells (HBMECs) treated with 100 μM H_2_O_2_ for 1–2 days, with or without CU06-1004 (1004), and then maintained in fresh media for 5 days. Representative microscopic images were captured with phase-contrast microscopy. **B** Quantification of SA-β-Gal–positive cells shown in **A**. **C**–**E** The relative mRNA levels of p16 and p21 in senescent HBMECs following exposure to H_2_O_2_ or CU06-1004 were quantified by reverse transcriptase–polymerase chain reaction. GAPDH, glyceraldehyde-3-phosphate dehydrogenase. All data were analyzed with one-way analysis of variance, followed by Tukey’s multiple comparison test. ^**###**^*P* < 0.001 vs. control. ***P < 0.001 vs. H_2_O_2_. n.s., not significant. Results are presented as the mean, and error bars represent the standard error of the mean

To investigate the effect of CU06-1004 treatment on HBMECs with a senescent phenotype, cells were treated with 100 µM H_2_O_2_ to induce cellular senescence (Fig. 3A). At 3 days post-H_2_O_2_ exposure, the presence of senescent HBMECs was confirmed by the presence of cells with an enlarged shape and cytoplasmic granularity. After 5 days, the SA-β-Gal staining assay was used to assess the level of senescence among HBMECs exposed to H_2_O_2_. A threefold increase in the percentage of SA-β-Gal^+^ cells was observed in H_2_O_2_-treated HBMECs compared with control cells. However, pretreatment with CU06-1004 significantly inhibited this effect and reduced the percentage of SA-β-Gal^+^ cells to twofold that of control cells (Fig. 3B). Senescence is a state of permanent cellular arrest that is established and maintained by the expression of cyclin-dependent kinase inhibitors. As p16^INK4a^ and p21 are known to mediate permanent cell cycle arrest [34, 35], we used RT-PCR to determine the mRNA levels associated with these genes. The expression levels of p16 ^INK4a^ and p21 were downregulated in the CU06-1004 treatment group, confirming the anti-senescence effect of CU06-1004 in HBMECs (Fig. 3C–E).

### CU06-1004 alleviates age-associated cerebral microvascular rarefaction and vascular aging in mice

**Fig. 4:**
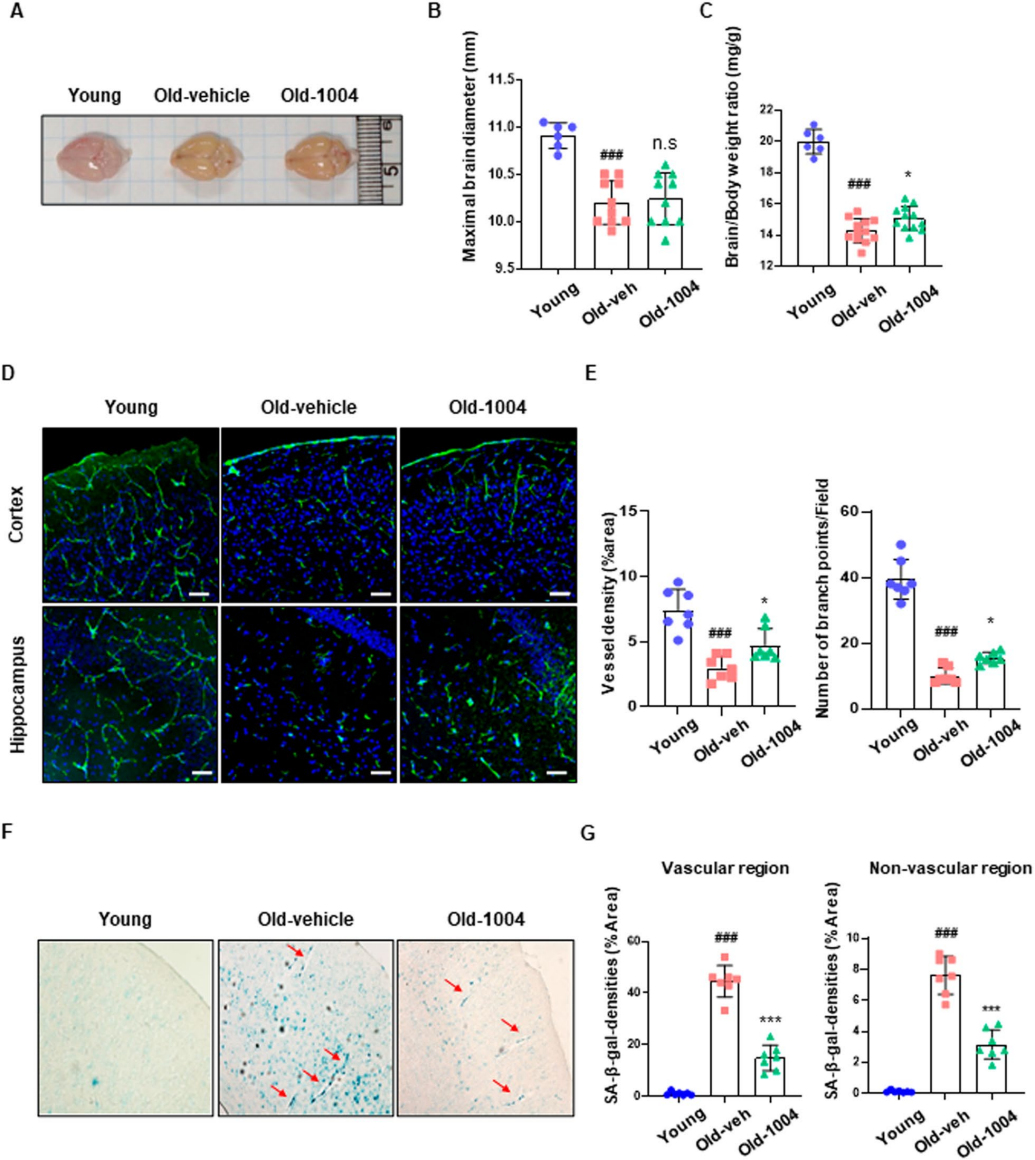
The effect of long-term CU06-1004 administration on cerebrovascular aging in mice. **A** Whole-mount view of brains from 6-week-old (young), 24-month-old vehicle-treated (old-veh), and 24-month-old CU06-1004–treated mice (old-1004) **B**, **C** Quantification of maximal brain diameter (**B**) and brain-to-body weight ratio (**C**, brain index) in young (n = 6), old-veh (n = 10–12), and old-1004 (n = 10–12) mice. **D**, **E** Representative images and quantification of capillary vessel density in the cortical and hippocampal regions of young, old-veh, and old-1004 mice. Scale bar = 50 µm. **F** Representative images of Senescence-associated β-galactosidase staining (SA-β-gal) in cortical regions of young, old-veh, and old-1004 mice (n = 7 per group). **G** Stained levels of SA β-gal-positive cells, digitized for analysis by Photoshop. Quantified SA-β-gal positive densities in vascular region was calculated as the ratio of the area covered by SA-β-gal positive vascular area to the total vascular area. And quantified SA-β-gal positive densities in non-vascular region was calculated as the ratio of the area covered by SA-β-gal positive non-vascular area to the total area excluding the vascular area. Interestingly, in the old-1004 group, SA-β-Gal^+^ cell prevalence was reduced in both vascular and non-vascular region, suggesting that CU06-1004 may alleviate cerebrovascular aging in both vascular and non-vascular cells (**G**). All data were analyzed with one-way analysis of variance, followed by Tukey’s multiple comparison test. ^**###**^*P* < 0.001 vs. young. *P < 0.05 vs. old-veh. Results are presented as the mean, and error bars represent the standard deviation

Based on results showing the anti-inflammatory and anti-senescence effects of CU06-1004 in HBMECs, we examined whether the long-term administration of CU06-1004 (10 mg/kg via oral gavage) to 18-month-old mice (late middle age) had protective effects against cerebrovascular aging. First, we measured the maximal cortical diameter and the brain-to-body weight ratio at the postmortem examination. The mean cortical diameter decreased by 8% in 24-month-old mice (late age) compared with 6-week-old mice (young age). However, among 24-month-old mice, no differences in cortical diameter were detected between those treated with vehicle (old-vehicle) and those treated with CU06-1004 (old-1004; Fig. 4A, B). We also measured the brain-to-body weight ratio because this measure serves as a rough estimate of intelligence and brain function in animals [36]. The relative brain-to-body weight ratio was significantly reduced in the old-vehicle group but was less reduced in the old-1004 group (Fig. 4C). The brains of CU06-1004-treated mice were heavier than the brains of vehicle-treated mice (Additional file 1: Figure S1). To examine age-related changes in the brain microvasculature, the patterning of the brain vasculature in the cortex and hippocampus was analyzed in the aged mouse brain through the immunofluorescent staining of endothelial markers. A significant reduction in capillary vessel density was observed for the microvasculature of old mice compared with the microvasculature of young mice. However, greater capillary vessel density and higher numbers of branch points were observed in the old-1004 group than in the old-vehicle group, suggesting that CU06-1004 improves vessel maintenance and inhibits cerebral microvascular rarefaction in aged mice (Fig. 4D, E). Additionally, SA-β-Gal^+^ cells were observed in vessels located in the cortex of aged mice, but the prevalence of SA-β-Gal^+^ cells decreased in the old-1004 group compared with the old-vehicle group (Fig. 4F). Interestingly, in the old-1004 group, SA-β-Gal^+^ cell prevalence was reduced in both vascular and non-vascular region, suggesting that CU06-1004 may alleviate cerebrovascular aging in both vascular and non-vascular cells (Fig. 4G). These results suggest that long-term administration of CU06-1004 prevents age-associated cerebral microvascular rarefaction and cerebrovascular aging.

### CU06-1004 treatment prevents the disruption of BBB integrity during aging

**Fig. 5:**
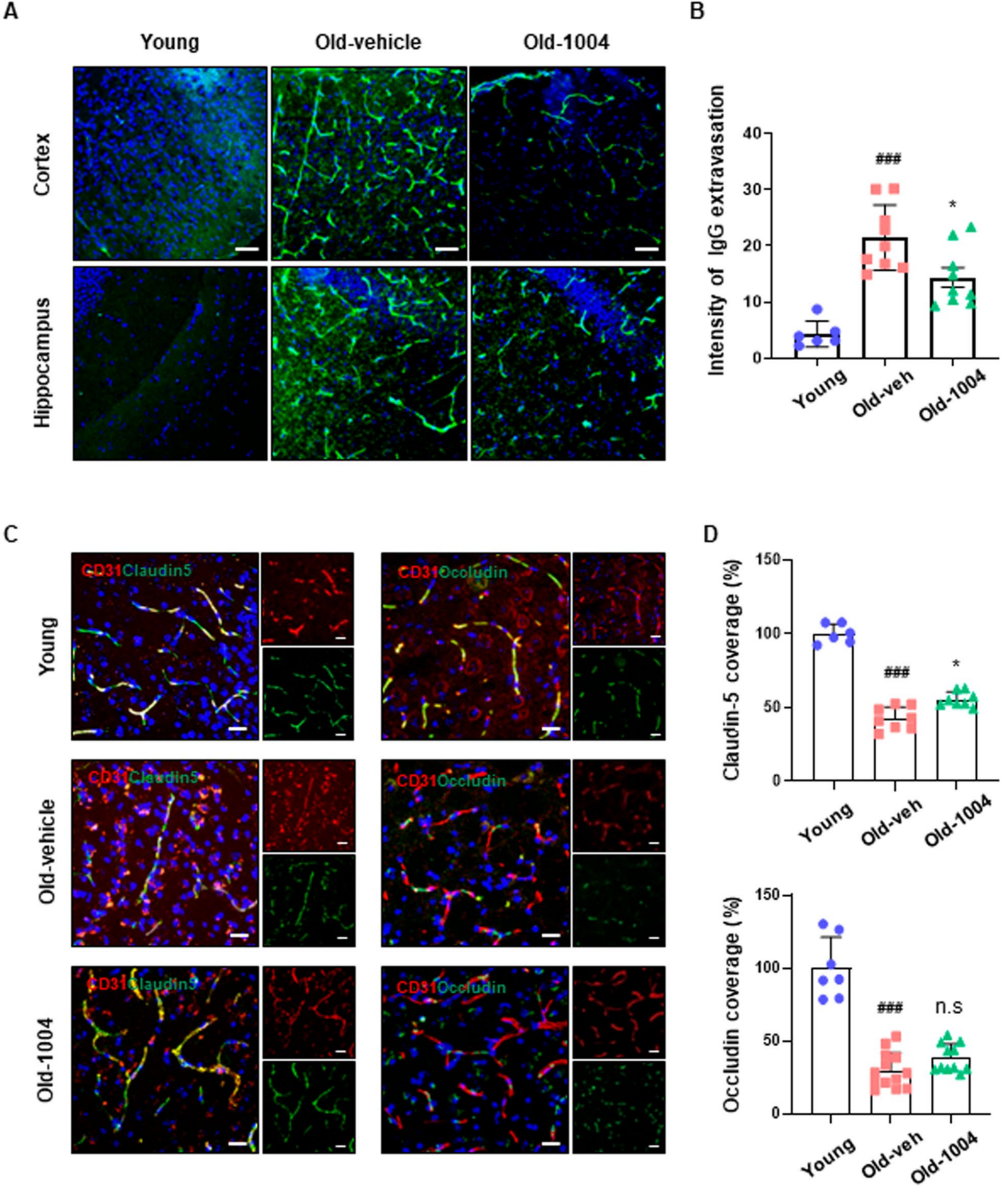
CU06-1004 administration rescued impaired blood–brain barrier integrity and reduced tight junction protein coverage in aged mice. **A** Confocal microscopic images of plasma IgG extravasation as a marker of blood–brain barrier disruption. Scale bar = 50 µm. **B** Quantitative analyses of mean IgG mean intensity in cortical and hippocampal regions in 6-week-old (young), 24-month-old vehicle-treated (old-veh), and 24-month-old CU06-1004–treated mice (old-1004). **C** Double immunostaining of claudin-5 and CD31 (left) and occludin and CD31 (right). Representative images show the abundance of claudin-5 (green) and occludin (green) in cerebral vessels of young, old-veh, and old-1004 mice. Scale bar = 20 µm. **D** Quantitative analysis of the claudin-5/CD31 and occludin/CD31 ratios (n = 6–10 per group). All data were analyzed with one-way analysis of variance, followed by Tukey’s multiple comparison test. ^**###**^*P* < 0.001 vs. young. *P < 0.05 vs. old-veh. n.s., not significant. Results are presented as the mean, and error bars represent the standard deviation

We then investigated whether age-induced cerebral microvascular rarefaction affects BBB integrity. BBB integrity was determined by detecting the cerebral extravasation of the plasma protein IgG. As shown in Fig. 5A, B, IgG was almost absent from the cortical and hippocampal parenchyma of young mice but was highly abundant in the parenchyma of aged mice. Notably, the IgG abundance was significantly decreased in the old-1004 group compared with the old-vehicle group. As BBB permeability is highly dependent on cerebrovascular endothelial tight junctions, we next examined tight junction integrity in the brains of young and aged mice. Compared with young mice, the cerebral vessels of aged mice expressed lower levels of the tight junction proteins claudin-5 and occludin, but claudin-5 was upregulated in aged mice that received CU06-1004 treatment (old-1004). However, no difference in occludin expression levels was observed between the aged groups (Fig. 5C, D). In addition, the protein expression levels of claudin-5 and occludin in aged mice brain tissues following old-1004, which revealed significantly increased protein expressions in old-1004 mice compared with old-vehicle (Additional file 2: Figure S2). We used electron microscopy to further examine the changes in tight junction complexes that might be responsible for cerebrovascular leakage. In young mice, ultrastructural analysis showed seamless tight junctions within a smooth endothelial layer surrounded by astrocytic end-feet. In aged mice, the capillary wall appeared thicker, and the astrocytic end-feet were considerably swollen; however, the tight junctional complex was less damaged in the old-1004 group than in the old-vehicle group (Additional file 3: Figure S3). These results indicate that aging accelerates the onset of BBB dysfunction, and the long-term administration of CU06-1004 might prevent damage to BBB integrity associated with aging.

### CU06-1004 attenuates neuropathological changes in the aged brain

**Fig. 6:**
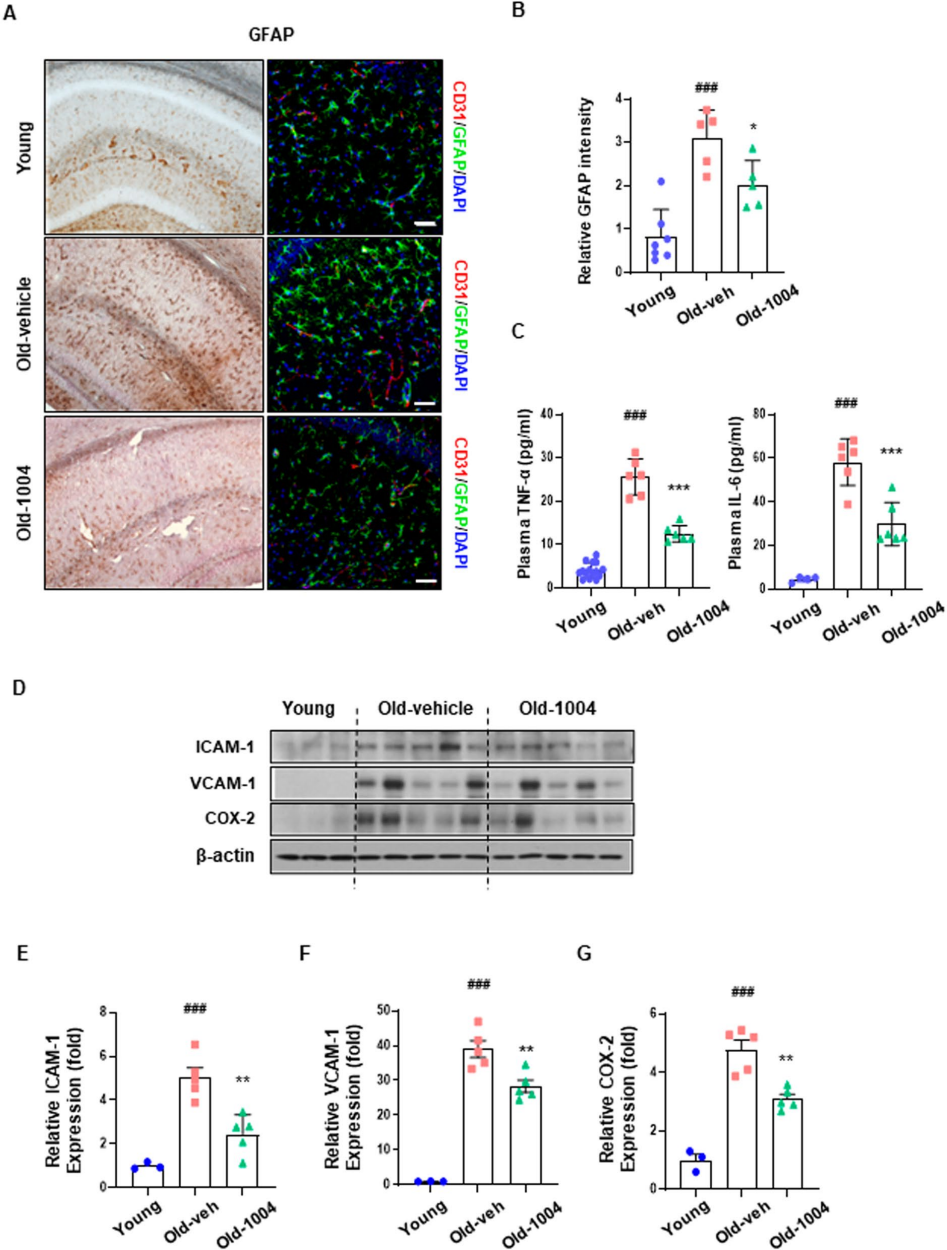
Effects of CU06-1004 on neuropathological changes in the aging brain. Aging affects the induction of astrocyte activation. **A** Histopathological analysis of astrocyte activation in the brains of 6-week-old (young), 24-month-old vehicle-treated (old-veh), and 24-month-old CU06-1004–treated mice (old-1004). Histopathological alterations were evaluated using immunohistochemistry (3,3′-diaminobenzidine [DAB]) and immunofluorescence staining. The cytoplasmic glial fibrillary acidic protein (GFAP)-positive astrocytes were observed in the hippocampus of all three groups. Double immunofluorescence showed increased GFAP-positive astrocytes in the hippocampus of aged mice compared with the hippocampus of young mice. Scale bar = 50 µm. **B** Astrocyte activation was quantified using fluorescent intensity. **C** Serum levels of tumor necrosis factor-alpha (TNF-α) and interleukin (IL)-6 in young, old-veh, and old-1004 mice. **D**–**G** Expression of inflammatory proteins in brain tissue extracts. β-actin was the internal control (n = 2–5 per group). ICAM-1, intracellular adhesion molecule 1; VCAM-1, vascular cell adhesion molecule 1; COX-2, cyclooxygenase 2. All data were analyzed with one-way analysis of variance, followed by Tukey’s multiple comparison test. ^**###**^*P* < 0.001 vs. young. *P < 0.05, **P < 0.01, ***P < 0.001 vs. old-veh. **B**, **C** Results are presented as the mean, and error bars represent the standard deviation. **E**–**G** Results are presented as the mean, and error bars represent the standard error of the mean

Studies have shown that BBB dysfunction amplifies and may act as a key process in the development of neuroinflammation [37]. Therefore, we evaluated astrocyte activation, a widely accepted neuroinflammatory hallmark, in the brain tissues of young and aged mice. Histopathological alterations were evaluated using immunohistochemistry and immunofluorescence staining. As shown in Fig. 6A, B, GFAP-positive (activated) astrocytes were significantly increased in hippocampal brain sections from aged mice compared with hippocampal brain sections from young mice. Double immunofluorescence staining showed increased GFAP-positive astrocytes in the hippocampal sections of aged mice, suggesting that aging causes the upregulation of activated astrocytes. Notably, the long-term administration of CU06-1004 reduced systemic aging-associated increases in TNF-α and IL-6 levels (Fig. 6C). Additionally, we analyzed expression levels of inflammatory proteins from brain tissue extracts. The expression levels of proteins, such as ICAM-1, VCAM-1, and COX-2, were lower in brain tissues from aged mice that received CU06-1004 (old-1004) than in untreated aged mice (old-vehicle; Fig. 6D–G). Collectively, these results indicate that the long-term administration of CU06-1004 exerts anti-inflammatory and neuroprotective effects in aged mice.

### CU06-1004 treatment, starting in late middle age, improves motor function and recognition memory dysfunction

**Fig. 7:**
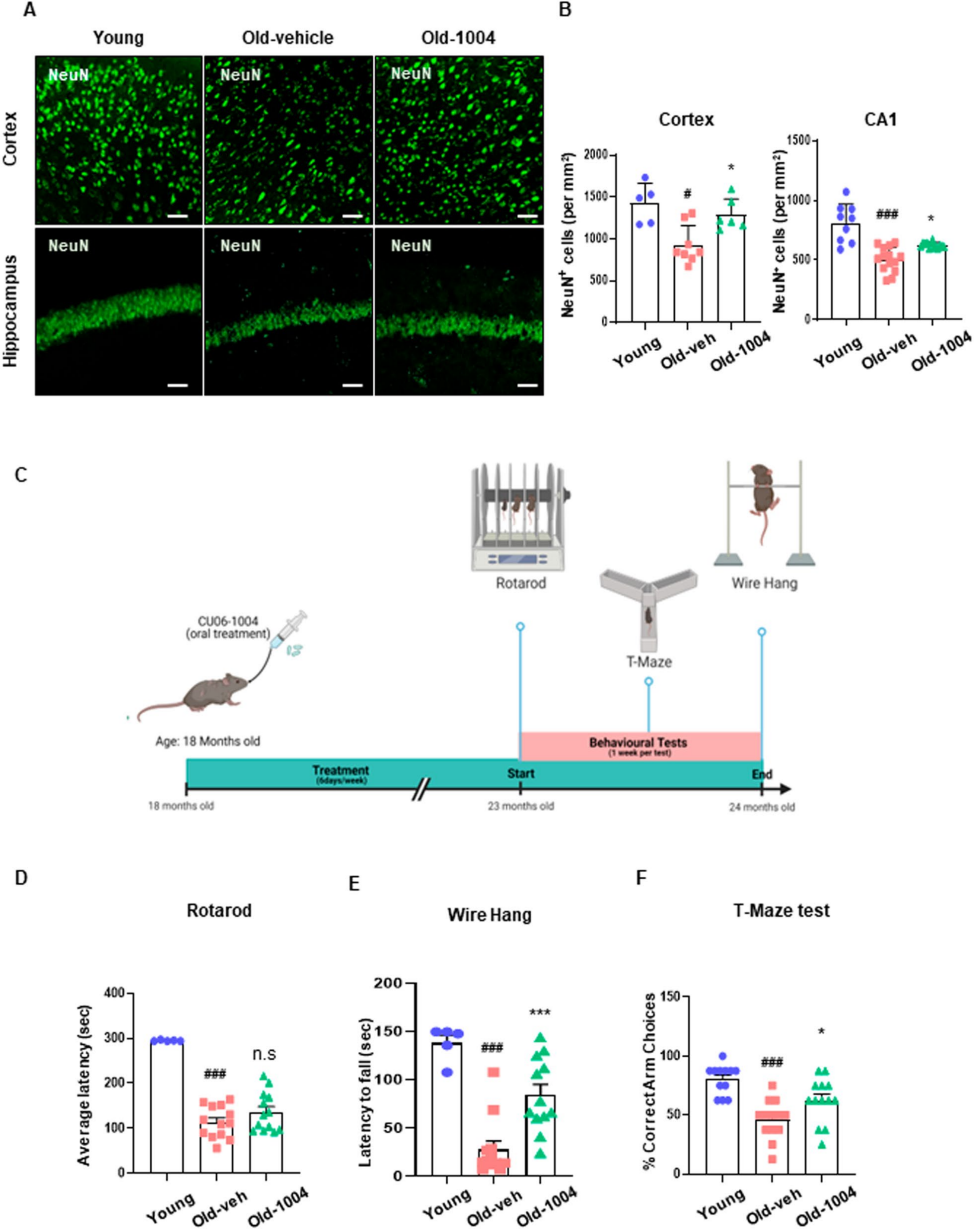
CU06-1004 reduces neuronal loss and cognitive deficits in aged mice. **A** Neuronal nuclei (NeuN) were visualized using immunofluorescence staining in brain tissues from 6-week-old (young), 24-month-old vehicle-treated (old-veh), and 24-month-old CU06-1004–treated mice (old-1004). Scale bar = 50 µm. **B** The number of NeuN-positive cells/mm^2^ was calculated in cortical and hippocampal CA1 regions in young, old-veh, and old-1004 mice. **C**–**F** Behavior tests were conducted on 23-month-old mice. **D** Rotarod test; **E** Wire hang test; **F** T-maze test. Mice were given oral injections of olive oil (vehicle) or CU06-1004 (10 mg/kg) for 6 months, and behavioral tests were performed separately at 23 months (n = 10–12 mice per group). ^**#**^*P* < 0.05, ^**###**^*P* < 0.001 vs. young. *P < 0.05, ***P < 0.001 vs. old-veh. **B** Results are presented as the mean, and error bars represent the standard deviation. **D**–**F** Results are presented as the mean, and error bars represent the standard error of the mean

Next, we quantified neuronal nuclear protein A60-positive (NeuN^+^) cells in the brains of aged mice. The number of NeuN^+^ cells was significantly reduced, and these cells were less compact in the cortical and hippocampal CA1 regions of the aged mouse brain than in the same regions of the young mouse brain. However, NeuN^+^ cell numbers and compactness were restored by CU06-1004 treatment (old-1004), demonstrating the efficacy of CU06-1004 in protecting against neuronal damage in the aged brain (Fig. 7A, B). We then examined whether aged mice treated with CU06-1004 showed behavioral and cognitive recovery (Fig. 7C). Motor function and working memory tests were performed with old-vehicle and old-1004 mice at 23 months. The rotarod test and wire hang test are classic methods for evaluating motor coordination and balance in aged animals. As shown in Fig. 7D, both the old-vehicle and old-1004 groups exhibited shorter average times to fall from the accelerating rotating rod than the young group, but no significant difference was detected between the old-vehicle and old-1004 groups. In the wire hang test, the old-1004 group showed a marked increase in hanging time (approximately threefold) compared with the average hanging time of the old-vehicle group, indicating that CU06-1004 enhances motor coordination and forelimb muscle strength (Fig. 7E). The T-maze test is a spontaneous alternation task for assessing spatial working memory. Aged mice demonstrated a significantly lower percentage of correct spontaneous alternation choices, indicating an impaired working memory. However, the old-1004 group displayed an increased percentage of correct spontaneous alternation choices compared with the old-vehicle group, indicating a significant effect of CU06-1004 treatment (Fig. 7F). These results suggest that the long-term administration of CU06-1004 reduces aging-associated neuromuscular strength impairments and damage to spatial working memory caused by neuronal cell damage.

## Discussion

Aging is a biological process characterized by the progressive deterioration of the structure and function of all organs over time [38]. Aging is also a major risk factor for developing various vascular diseases, including cardiovascular diseases, stroke, eye diseases, and neurodegenerative diseases. The vascular system, which supplies oxygen and nutrients throughout the body, is affected by the aging process and becomes more susceptible to disease in the aged population. Therefore, the development of novel therapies capable of slowing the aging process and effectively treating aging-related diseases is critical [39].

CU06-1004, an endothelial cell dysfunction blocker, prevents vascular leakage, enhances vascular integrity in ischemic reperfusion injury, and promotes the normalization of tumor vasculature. However, the mechanisms underlying the roles played by CU06-1004 in oxidative stress–induced HBMEC senescence, inflammation, and age-related cerebrovascular dysfunction remain unknown. In this study, the brains of aged mice showed higher SA-β-Gal activity than the brains of young mice. The brain capillaries of young mice appeared as interconnected, tubular structures, whereas the brain capillaries of aged mice appeared fragmented and disconnected in both the cortical and hippocampal regions. These changes in capillary structures suggest that BMECs have become senescent, a state of irreversible cell growth inhibition known to contribute to the decrease in cerebral capillary density observed during aging (Fig. 4). Mice 18–24 months of age are used to represent humans 56–69 years of age. Between the ages of 55 and 85 years, human brain tissue has been characterized as showing significant decreases in microvasculature density, similar to the decreases observed in Alzheimer’s disease patients [40]. In normal aging, cerebrovascular loss results in chronic brain hypoperfusion, eventually leading to cognitive impairment and vascular dementia [40]. Recent studies have shown that cerebrovascular disease is among the factors that play important roles in Alzheimer’s disease development. In particular, cerebrovascular density during normal aging may lead to neuronal apoptosis, contributing to neurodegeneration [41]. Therefore, maintaining cerebrovascular homeostasis is important for preventing cerebrovascular aging and brain pathology. Moreover, we observed that cerebral microvascular rarefaction in aged brain tissue is associated with impaired BBB integrity, which, in turn, leads to exceedingly high trans-endothelial permeability and increased passive extravasation of plasma IgG [42, 43].

Here, we show that the long-term administration of CU06-1004 to aged mice alleviates age-associated cerebral microvascular rarefaction and inhibits the leakage of plasma IgG into the brain parenchyma by suppressing cellular senescence and upregulating the stability of claudin-5, the most enriched tight junction protein in the aged mouse brain (Fig. 5).

BBB integrity is also strongly affected by oxidative stress, and increased ROS production contributes to cerebral endothelium dysfunction and increased BBB permeability [44]. Additionally, cerebral endothelial cells have high concentrations of mitochondria, increasing the risks of cellular oxidative damage [45]. The oxidation–inflammation theory of aging also proposes that age-associated oxidative stress is a driving factor in cellular senescence [11]. Consistent with previous studies, we observed that the H_2_O_2_-induced generation of excessive free radicals activated HBMEC senescence, resulting in cells exhibiting classical SASP characteristics, such as an enlarged cell shape, cytoplasmic granularity, and increased SA-β-Gal activity. H_2_O_2_ exposure activated cell cycle inhibition pathways, including p16^INK4a^ and p21, and strongly suppressed cell proliferative capacity. By contrast, HBMECs supplemented with CU06-1004 were characterized by attenuated SA-β-Gal activity and the marked downregulation of inflammatory proteins associated with SASP, potentially due to NF-κB inhibition. Additionally, CU06-1004 treatment appeared to prevent senescence-associated cell cycle arrest by inhibiting the cell cycle suppressors p16^INK4a^ and p21. HBMECs treated with CU06-1004 showed improved proliferative capacity following H_2_O_2_ exposure compared with control cells (Fig. 3). Overall, these results indicate that CU06-1004 inhibits the development of oxidative stress–induced senescence-associated features and the inflammatory response in HBMECs.

Increased chronic systemic inflammation during aging results in increased proinflammatory cytokines and other factors that damage the cerebrovasculature [46–49]. Chronic systemic inflammation, a type of low-grade, persistent inflammation, causes tissue degeneration. Additionally, chronic, low-grade inflammation contributes to various age-related pathologies in the tissues of the nervous and musculoskeletal systems [31, 50, 51]. We found that long-term CU06-1004 administration reduced systemic inflammation due to increased plasma concentrations of proinflammatory cytokines, such as TNF-α and IL-6. These results suggest that in addition to protecting against vascular damage, CU06-1004 may also inhibit inflammation in the brain and other tissues a (Fig. 6). In this study, we did not directly investigate the protective effects of CU06-1004 in tissues other than the brain. However, we observed improved motor function and recognition memory in aged mice receiving long-term CU06-1004 administration (Fig. 7). A prior study demonstrated that changes in structure and function due to aging result in decreased capillary densities in other tissues, reducing blood flow to muscles and affecting exercise performance [52]. Therefore, we suggest that long-term administration of CU06-1004 may enhance exercise capacity by not only affecting the cerebrovasculature but also improving blood flow to muscles. These findings emphasize the importance of the BBB in maintaining the normal function of the central nervous system, resisting neuronal injury, and improving cognitive function (Additional file 4: Fig. S4).

## Conclusions

In conclusion, this study demonstrated that cerebrovascular aging might contribute to age-related cerebrovascular damage and neuroinflammation. In HBMECs, the endothelial cells found in cerebral blood vessels, treatment with CU06-1004, a known endothelial dysfunction blocker, was able to protect against oxidative stress–induced senescence and inflammation through ROS scavenging, leading to reduced cytotoxicity. Long-term administration of CU06-1004 in aged mice alleviated motor and cognitive deficits and associated cerebral damage, including cerebral microvascular rarefaction, neuronal losses, and chronic neuroinflammation, together with improved BBB integrity. Collectively, these results suggest that CU06-1004 could represent a useful therapeutic strategy for preventing cerebrovascular aging and age-associated brain injury.

## Supplementary Information

**Additional file 1: Figure S1. **Brain and body weight in aged mice. Brain and body weight of old-vehicle and old-1004 mice at 24 months of age. (A) Brain weight (n = 12 per group). (B) Body weight (n = 12 per group). All data were analyzed with unpaired two-tailed *t* test. *P < 0.05 vs. old-veh. n.s., not significant. Results are presented as the mean, and error bars represent the standard deviation.

**Additional file 2: Figure S2. **The protein levels of tight junctions in aged mice brain. Western blotting analysis was used to assess the protein expression levels of claudin-5 and occludin in aged mouse brain. (A-B) The protein levels of claudin-5 and occludin in aged mice brain tissue extracts. β-actin was the internal control (n = 5 per group). All data were analyzed with unpaired two-tailed *t* test. **P < 0.01, ***P < 0.001 vs. old-veh. Results are presented as the mean, and error bars represent the standard error of the mean.

**Additional file 3: Figure S3. **Electron micrographic images of cerebrovasculature in young and aged mice. Transmission electron microscopy (TEM) was used to observe the blood–brain barrier (BBB) ultrastructure in 6-week-old (young), 24-month-old vehicle-treated (old-veh), and 24-month-old CU06-1004–treated mice (old-1004). EC; endothelial cell, VL; vessel lumen, TJ; tight junction, AC; astrocyte, PC; pericyte. Black arrows indicate brain endothelial TJs. (A–B) Intact BBB in blood vessels (VL) embedded in closed TJs between brain endothelial cells of young mice. Scale bar = 2 µm. (A1–B1) High-magnification images of the red-boxed areas in A and B, highlighting endothelial TJs with black arrows. Scale bar = 500 nm. (C–F) Disrupted BBB in blood vessels (VL), including thicker capillary walls and swollen astrocytic end-feet in old-veh and old-1004 mice. Scale bar = 2 µm. (C1–F1) High-magnifications images of the red-boxed areas in C–F showing discontinuous and increased gaps between cerebrovascular TJs, reflecting a disrupted BBB in old-veh and old-1004 mice. Scale bar = 500 nm. (G) Junctional complex average width (nm) was quantitatively analyzed by measuring the average width between TJs in TEM images from young, old-veh, and old-1004 mice (n = 9–12 per group). Red bars mean tight junctional complex average width between tight junction complexes. ^###^*P* < 0.001 vs. young. ***P < 0.001 vs. old-veh. Results are presented as the mean, and error bars represent the standard deviation.

**Additional file 4: Figure S4.** Uncropped Image.

## Data Availability

Not applicable.

## Abbreviations

BBB: Blood–brain barrier
BMEC: Brain microvascular endothelial cell
CD31: Cluster of differentiation 31
COX-2: Cyclooxygenase-2
DMSO: Dimethyl sulfoxide
EGM-2: Endothelial cell growth medium 2
GAPDH: Glyceraldehyde-3-phosphate dehydrogenase
GFAP: Glial fibrillary acidic protein
H_2_-DCFDA: 2′,7′-Dichlorodihydrofluorescein diacetate
H_2_O_2_: Hydrogen peroxide
HBMEC: Human brain microvascular endothelial cell
ICAM-1: Intercellular adhesion molecule 1
IgG: Immunoglobulin G
IkBα: Nuclear factor of kappa light polypeptide gene enhancer in B cells inhibitor, alpha
IL-6: Interleukin 6
MTT: 3-(4,5-Dimetylthialzol-2-yl)-2,5-diphenyltertrazolium bromide
NeuN: Neuronal nuclear protein
NF-ĸB: Nuclear factor-kappa B
OCT: Optimal cutting temperature
PBS: Phosphate-buffered saline
ROS: Reactive oxygen species
RT: Room temperature
RT-PCR: Reverse transcriptase–polymerase chain reaction
SA-β-Gal: Senescence-associated β-galactosidase
SASP: Senescence-associated secretory phenotype
TBST: Tris-buffered saline containing Tween-20
TNF-α: Tumor necrosis factor-alpha
VCAM-1: Vascular adhesion molecule-1
