# Supplementary figures and images for: Long-term administration of CU06-1004 ameliorates cerebrovascular aging and BBB injury in aging mouse model
Source: Fluids Barriers CNS. 2023 Feb 1;20:9. doi: 10.1186/s12987-023-00410-x (PMC9893613; doi:10.1186/s12987-023-00410-x)

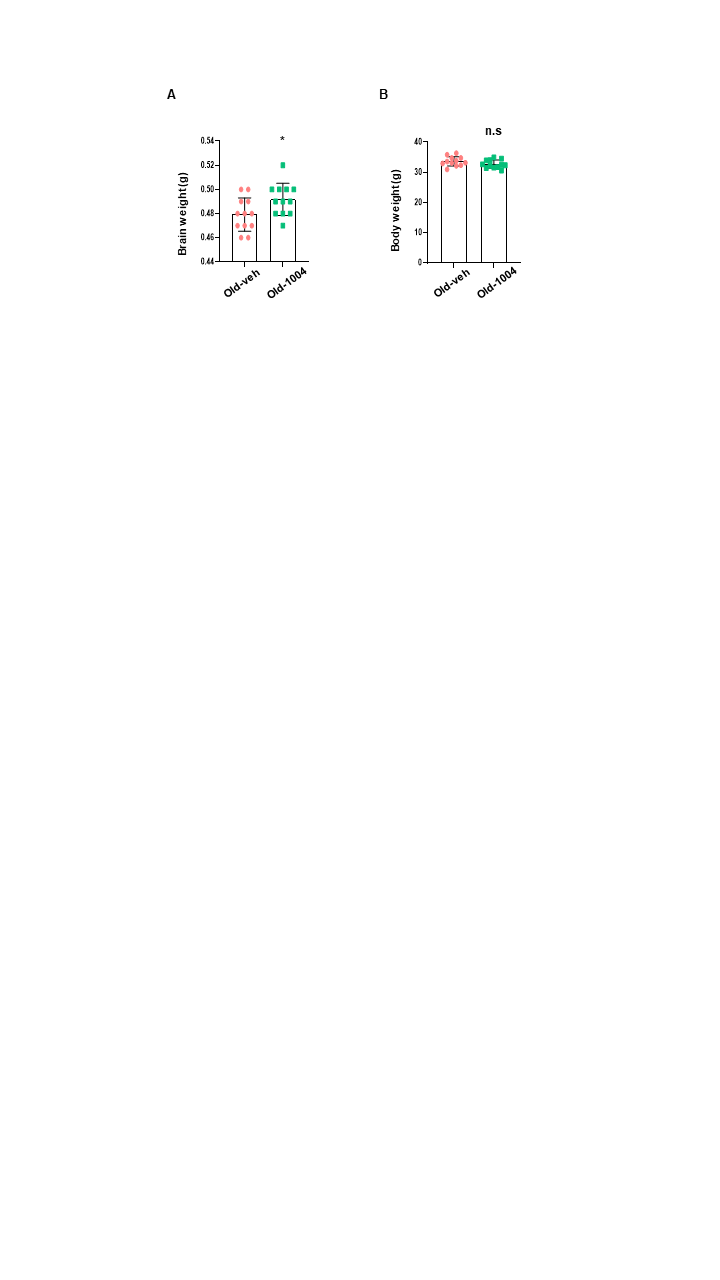

Supplement: Supplementary file 1 — Additional file 1: Figure S1. Brain and body weight in aged mice. Brain and body weight of old-vehicle and old-1004 mice at 24 months of age. (A) Brain weight (n = 12 per group). (B) Body weight (n = 12 per group). All data were analyzed with unpaired two-tailed t test. *P < 0.05 vs. old-veh. n.s., not significant. Results are presented as the mean, and error bars represent the standard deviation. [file 12987_2023_410_MOESM1_ESM.tif]

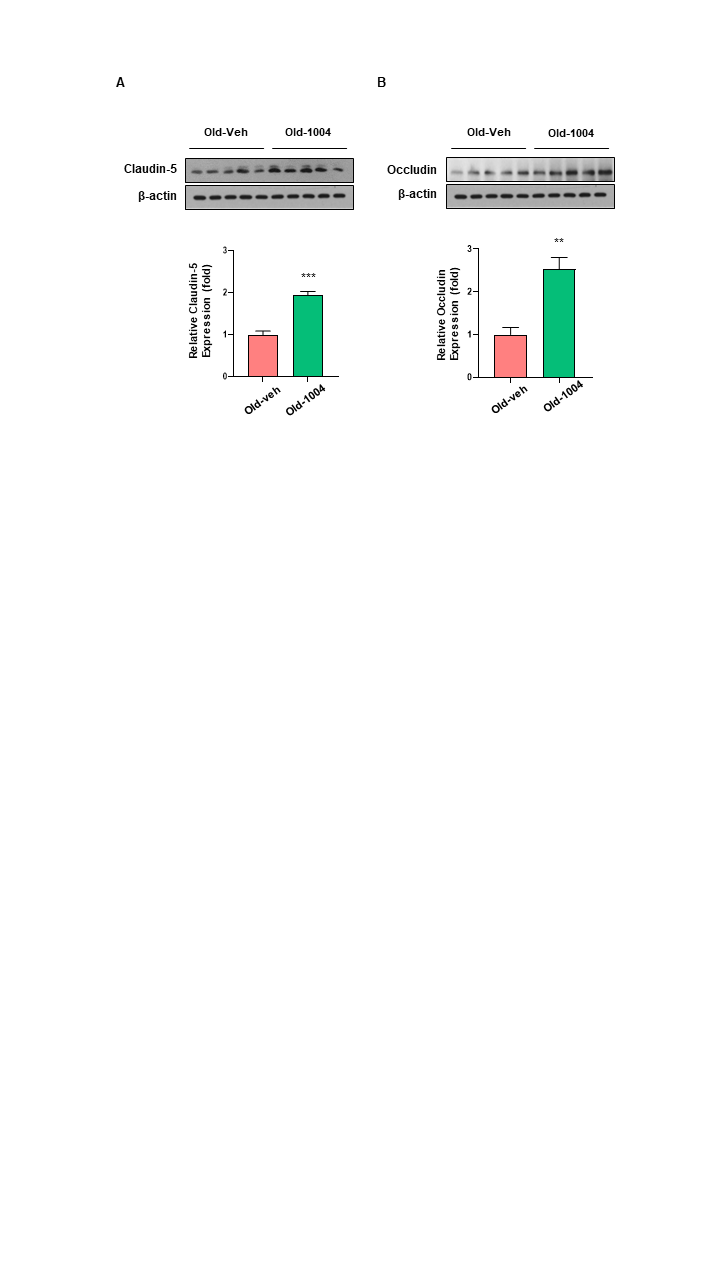

Supplement: Supplementary file 2 — Additional file 2: Figure S2. The protein levels of tight junctions in aged mice brain. Western blotting analysis was used to assess the protein expression levels of claudin-5 and occludin in aged mouse brain. (A-B) The protein levels of claudin-5 and occludin in aged mice brain tissue extracts. β-actin was the internal control (n = 5 per group). All data were analyzed with unpaired two-tailed t test. **P < 0.01, ***P < 0.001 vs. old-veh. Results are presented as the mean, and error bars represent the standard error of the mean. [file 12987_2023_410_MOESM2_ESM.tif]

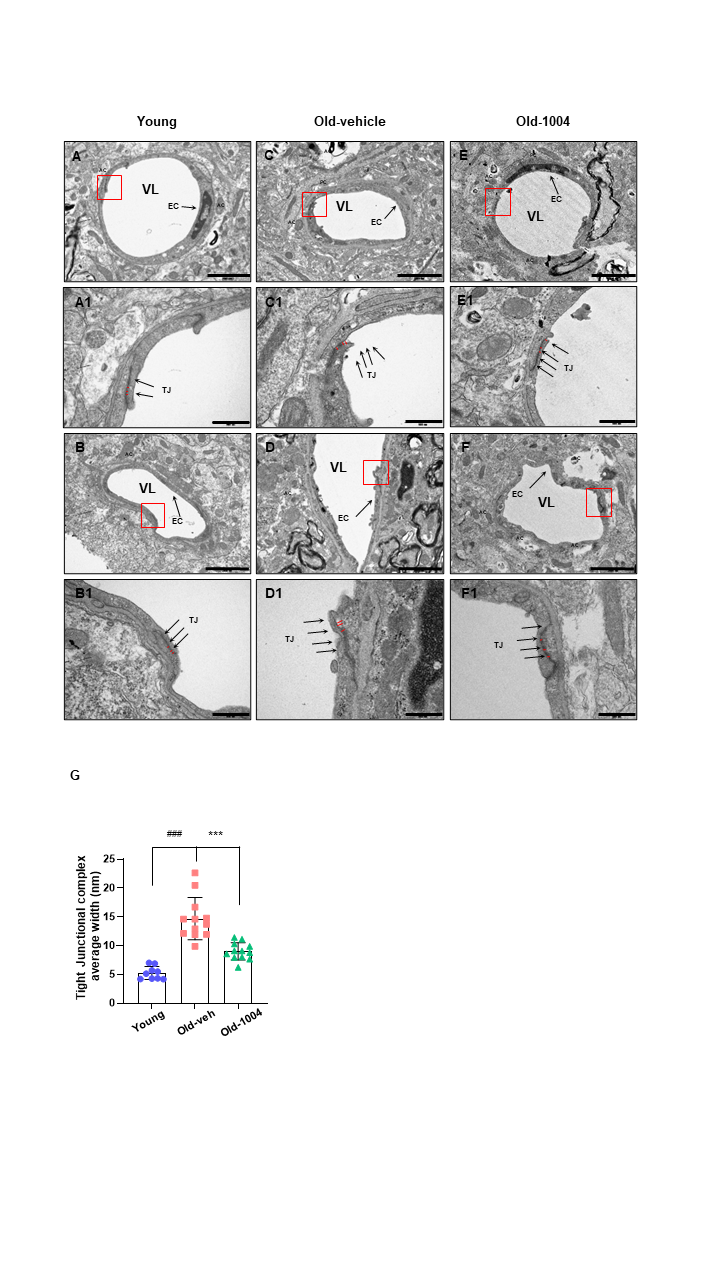

Supplement: Supplementary file 3 — Additional file 3: Figure S3. Electron micrographic images of cerebrovasculature in young and aged mice. Transmission electron microscopy (TEM) was used to observe the blood–brain barrier (BBB) ultrastructure in 6-week-old (young), 24-month-old vehicle-treated (old-veh), and 24-month-old CU06-1004–treated mice (old-1004). EC; endothelial cell, VL; vessel lumen, TJ; tight junction, AC; astrocyte, PC; pericyte. Black arrows indicate brain endothelial TJs. (A–B) Intact BBB in blood vessels (VL) embedded in closed TJs between brain endothelial cells of young mice. Scale bar = 2 µm. (A1–B1) High-magnification images of the red-boxed areas in A and B, highlighting endothelial TJs with black arrows. Scale bar = 500 nm. (C–F) Disrupted BBB in blood vessels (VL), including thicker capillary walls and swollen astrocytic end-feet in old-veh and old-1004 mice. Scale bar = 2 µm. (C1–F1) High-magnifications images of the red-boxed areas in C–F showing discontinuous and increased gaps between cerebrovascular TJs, reflecting a disrupted BBB in old-veh and old-1004 mice. Scale bar = 500 nm. (G) Junctional complex average width (nm) was quantitatively analyzed by measuring the average width between TJs in TEM images from young, old-veh, and old-1004 mice (n = 9–12 per group). Red bars mean tight junctional complex average width between tight junction complexes. ###P < 0.001 vs. young. ***P < 0.001 vs. old-veh. Results are presented as the mean, and error bars represent the standard deviation. [file 12987_2023_410_MOESM3_ESM.tif]

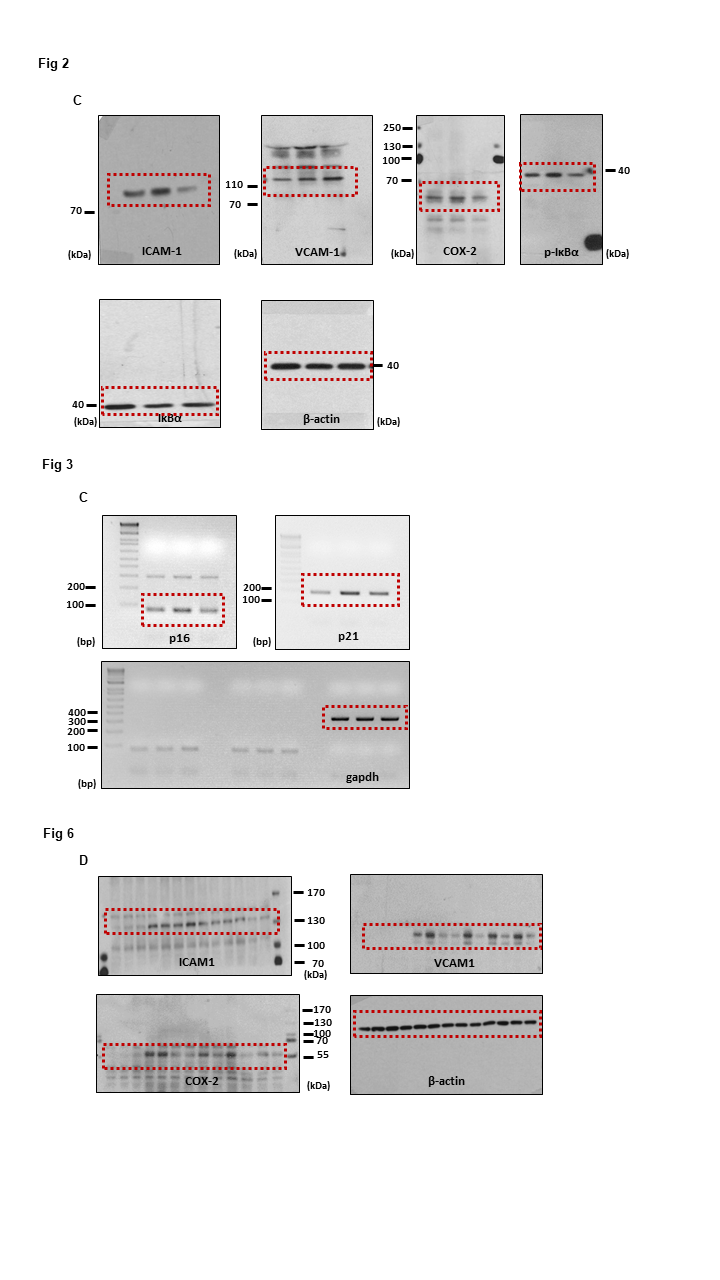

Supplement: Supplementary file 4 — Additional file 4: Figure S4. Uncropped Image. [file 12987_2023_410_MOESM4_ESM.tif]
